# (*E*)-1-(4-Methyl­phen­yl)-3-[(1-phenyl­ethyl­idene)amino]­thio­urea

**DOI:** 10.1107/S1600536811019404

**Published:** 2011-05-28

**Authors:** Yan-Ling Zhang, Chang-Zeng Wu, Fu-Juan Zhang

**Affiliations:** a College of Chemistry and Chemical Engineering, Xuchang University, Henan 461000, People’s Republic of China

## Abstract

In the title compound, C_16_H_17_N_3_S, the amino­thio­urea unit is nearly planar (r.m.s. deviation = 0.0425 Å), and is twisted with respect to the tolyl and phenyl rings by 57.84 (7) and 15.88 (14)°, respectively; the tolyl and phenyl rings are twisted by 65.64 (11)° to each other. Inter­molecular N—H⋯S and weak C—H⋯S hydrogen bonds are present in the crystal structure.

## Related literature

The title compound is a derivative of thio­semicarbazone. For applications of thio­semicarbazones in the biological field, see: Hu *et al.* (2006 ▶).
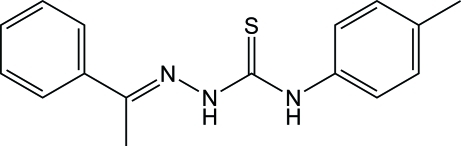


## Experimental

### 

#### Crystal data

C_16_H_17_N_3_S
*M* *_r_* = 283.39
Monoclinic, 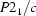

*a* = 10.5881 (3) Å
*b* = 5.7355 (2) Å
*c* = 26.9746 (7) Å
β = 108.670 (2)°
*V* = 1551.91 (8) Å^3^
*Z* = 4
Cu *K*α radiation
μ = 1.79 mm^−1^
*T* = 291 K
0.25 × 0.18 × 0.18 mm

#### Data collection

Oxford Diffraction Xcalibur Eos Gemini diffractometer
Absorption correction: multi-scan (*CrysAlis PRO*; Oxford Diffraction, 2010 ▶) *T*_min_ = 0.60, *T*_max_ = 0.73
15835 measured reflections
2945 independent reflections
2587 reflections with *I* > 2σ(*I*)
*R*_int_ = 0.041

#### Refinement

*R*[*F* ^2^ > 2σ(*F* ^2^)] = 0.044
*wR*(*F* ^2^) = 0.126
*S* = 1.06
2945 reflections
191 parameters
H atoms treated by a mixture of independent and constrained refinement
Δρ_max_ = 0.23 e Å^−3^
Δρ_min_ = −0.24 e Å^−3^

### 

Data collection: *CrysAlis PRO* (Oxford Diffraction, 2010 ▶); cell refinement: *CrysAlis PRO*; data reduction: *CrysAlis PRO*; program(s) used to solve structure: *SHELXTL* (Sheldrick, 2008 ▶); program(s) used to refine structure: *SHELXTL*; molecular graphics: *OLEX2* (Dolomanov *et al.*, 2009 ▶); software used to prepare material for publication: *OLEX2*.

## Supplementary Material

Crystal structure: contains datablocks I, global. DOI: 10.1107/S1600536811019404/xu5214sup1.cif

Structure factors: contains datablocks I. DOI: 10.1107/S1600536811019404/xu5214Isup2.hkl

Supplementary material file. DOI: 10.1107/S1600536811019404/xu5214Isup3.cml

Additional supplementary materials:  crystallographic information; 3D view; checkCIF report

## Figures and Tables

**Table 1:** Hydrogen-bond geometry (Å, °)

*D*—H⋯*A*	*D*—H	H⋯*A*	*D*⋯*A*	*D*—H⋯*A*
N2—H2⋯S1^i^	0.90 (2)	2.86 (2)	3.7456 (16)	167.8 (19)
C16—H16*B*⋯S1^i^	0.96	2.74	3.471 (2)	133

Symmetry code: (i) 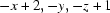.

**Fig. 1.**
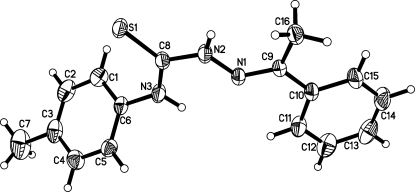
The molecular structure of the title compound, with displacement ellipsoids drawn at 30% probability level.

## supplementary crystallographic information

### Comment

Thiosemicarbazones have attracted our attention because of their biological applications (Hu *et al.*, 2006). A few single-crystal structures were reported. For understanding their anticancer activity, it is necessary to have detailed information on their geometries.

The molecular structure of (I) is shown in Fig 1. The molecules reveal an E configuration. The dihedral angles formed by the tolyl and phenyl rings with the almost planar aminothiourea unit (r.m.s. deviation = 0.0425 Å) are 57.84 (7) and 15.88 (14)°, respectively. Intermolecular N—H···S and C—H···S interactions occur in the crystal structure (Table 1).

### Experimental

*N*-(*p*-Tolyl)thiosemicarbazide (2.7 g, 15 mmol) and acetophenone (1.8 g, 15 mmol) was dissolved in 95% ethanol (20 ml), and the solution was refluxed for 3 h. Fine colorless crystals appeared on cooling. They were filtered and washed by 95% ethanol to give 2.9 g of the title compound in 69.0% yield. Single crystals suitable for X-ray measurements were obtained from ether by slow evaporation at room temperature.

### Refinement

Imino H atoms were located in a difference Fourier map and refined isotropically. Other H atoms were placed in calculated positions with C—H = 0.93–0.96 and refined using a riding model, *U*_iso_(H) = 1.5*U*_eq_(C) for methyl H atoms and 1.2*U*_eq_(C) for the others.

### Crystal data

**Table:**

C_16_H_17_N_3_S	*F*(000) = 600
*M**_r_* = 283.39	*D*_x_ = 1.213 Mg m^−^^3^
Monoclinic, *P*2_1_/*c*	Cu *K*α radiation, λ = 1.5418 Å
*a* = 10.5881 (3) Å	Cell parameters from 6724 reflections
*b* = 5.7355 (2) Å	θ = 3.5–70.9°
*c* = 26.9746 (7) Å	µ = 1.79 mm^−^^1^
β = 108.670 (2)°	*T* = 291 K
*V* = 1551.91 (8) Å^3^	Prismatic, colorless
*Z* = 4	0.25 × 0.18 × 0.18 mm

### Data collection

**Table:**

Oxford Diffraction Xcalibur Eos Gemini diffractometer	2945 independent reflections
Radiation source: Enhance (Cu) X-ray Source	2587 reflections with *I* > 2σ(*I*)
graphite	*R*_int_ = 0.041
Detector resolution: 16.2312 pixels mm^-1^	θ_max_ = 70.9°, θ_min_ = 3.5°
ω scans	*h* = −12→12
Absorption correction: multi-scan (*CrysAlis PRO*; Oxford Diffraction, 2010)	*k* = −7→6
*T*_min_ = 0.60, *T*_max_ = 0.73	*l* = −28→31
15835 measured reflections	

### Refinement

**Table:**

Refinement on *F*^2^	Primary atom site location: structure-invariant direct methods
Least-squares matrix: full	Secondary atom site location: difference Fourier map
*R*[*F*^2^ > 2σ(*F*^2^)] = 0.044	Hydrogen site location: inferred from neighbouring sites
*wR*(*F*^2^) = 0.126	H atoms treated by a mixture of independent and constrained refinement
*S* = 1.06	*w* = 1/[σ^2^(*F*_o_^2^) + (0.0693*P*)^2^ + 0.2489*P*] where *P* = (*F*_o_^2^ + 2*F*_c_^2^)/3
2945 reflections	(Δ/σ)_max_ = 0.001
191 parameters	Δρ_max_ = 0.23 e Å^−^^3^
0 restraints	Δρ_min_ = −0.24 e Å^−^^3^

### Special details

**Table:**

Geometry. All e.s.d.'s (except the e.s.d. in the dihedral angle between two l.s. planes) are estimated using the full covariance matrix. The cell e.s.d.'s are taken into account individually in the estimation of e.s.d.'s in distances, angles and torsion angles; correlations between e.s.d.'s in cell parameters are only used when they are defined by crystal symmetry. An approximate (isotropic) treatment of cell e.s.d.'s is used for estimating e.s.d.'s involving l.s. planes.
Refinement. Refinement of *F*^2^ against ALL reflections. The weighted *R*-factor *wR* and goodness of fit *S* are based on *F*^2^, conventional *R*-factors *R* are based on *F*, with *F* set to zero for negative *F*^2^. The threshold expression of *F*^2^ > σ(*F*^2^) is used only for calculating *R*-factors(gt) *etc*. and is not relevant to the choice of reflections for refinement. *R*-factors based on *F*^2^ are statistically about twice as large as those based on *F*, and *R*- factors based on ALL data will be even larger.

### Fractional atomic coordinates and isotropic or equivalent isotropic displacement parameters (Å^2^)

**Table:**

	*x*	*y*	*z*	*U*_iso_*/*U*_eq_	
S1	1.08154 (5)	0.02598 (9)	0.436028 (18)	0.05334 (18)	
N1	0.82552 (14)	0.5213 (3)	0.42960 (6)	0.0465 (3)	
N2	0.90859 (14)	0.3341 (3)	0.44695 (6)	0.0499 (4)	
N3	0.99833 (16)	0.4238 (3)	0.38311 (6)	0.0510 (4)	
C1	1.06088 (19)	0.2091 (4)	0.31610 (7)	0.0559 (5)	
H1	1.0044	0.0876	0.3179	0.067*	
C2	1.1318 (2)	0.1997 (4)	0.28098 (7)	0.0656 (6)	
H2A	1.1229	0.0699	0.2595	0.079*	
C3	1.2149 (2)	0.3777 (5)	0.27725 (7)	0.0651 (6)	
C4	1.2278 (2)	0.5681 (4)	0.30964 (8)	0.0630 (5)	
H4	1.2841	0.6896	0.3077	0.076*	
C5	1.15789 (19)	0.5810 (4)	0.34519 (7)	0.0520 (4)	
H5	1.1672	0.7109	0.3667	0.062*	
C6	1.07490 (16)	0.4014 (3)	0.34853 (6)	0.0443 (4)	
C7	1.2928 (3)	0.3658 (8)	0.23886 (12)	0.1137 (13)	
H7A	1.2491	0.4593	0.2087	0.171*	
H7B	1.2971	0.2070	0.2283	0.171*	
H7C	1.3814	0.4237	0.2553	0.171*	
C8	0.99248 (15)	0.2719 (3)	0.42007 (6)	0.0440 (4)	
C9	0.74014 (16)	0.5714 (3)	0.45287 (7)	0.0437 (4)	
C10	0.64955 (15)	0.7680 (3)	0.43048 (6)	0.0452 (4)	
C11	0.6772 (2)	0.9235 (4)	0.39573 (7)	0.0584 (5)	
H11	0.7547	0.9058	0.3869	0.070*	
C12	0.5910 (3)	1.1032 (5)	0.37429 (9)	0.0761 (7)	
H12	0.6102	1.2057	0.3510	0.091*	
C13	0.4749 (2)	1.1320 (5)	0.38739 (9)	0.0783 (8)	
H13	0.4162	1.2526	0.3726	0.094*	
C14	0.4478 (2)	0.9828 (5)	0.42192 (12)	0.0773 (7)	
H14	0.3707	1.0024	0.4309	0.093*	
C15	0.53386 (19)	0.8031 (4)	0.44367 (10)	0.0665 (6)	
H15	0.5146	0.7037	0.4675	0.080*	
C16	0.7269 (2)	0.4397 (4)	0.49895 (8)	0.0570 (5)	
H16A	0.6858	0.5377	0.5182	0.086*	
H16B	0.8137	0.3932	0.5212	0.086*	
H16C	0.6728	0.3038	0.4869	0.086*	
H2	0.904 (2)	0.230 (4)	0.4714 (8)	0.058 (6)*	
H3	0.960 (2)	0.548 (4)	0.3831 (8)	0.053 (6)*	

### Atomic displacement parameters (Å^2^)

**Table:**

	*U*^11^	*U*^22^	*U*^33^	*U*^12^	*U*^13^	*U*^23^
S1	0.0594 (3)	0.0488 (3)	0.0626 (3)	0.01893 (19)	0.0347 (2)	0.01199 (19)
N1	0.0428 (7)	0.0442 (9)	0.0593 (8)	0.0093 (6)	0.0257 (6)	0.0052 (6)
N2	0.0502 (8)	0.0488 (9)	0.0607 (8)	0.0150 (7)	0.0319 (6)	0.0117 (7)
N3	0.0550 (8)	0.0492 (10)	0.0578 (8)	0.0193 (7)	0.0305 (7)	0.0099 (7)
C1	0.0614 (10)	0.0575 (12)	0.0464 (8)	0.0029 (9)	0.0138 (7)	−0.0037 (8)
C2	0.0828 (13)	0.0722 (15)	0.0404 (8)	0.0195 (11)	0.0177 (8)	−0.0086 (8)
C3	0.0717 (12)	0.0870 (17)	0.0436 (9)	0.0292 (12)	0.0282 (8)	0.0116 (9)
C4	0.0648 (11)	0.0709 (14)	0.0624 (11)	0.0067 (10)	0.0333 (9)	0.0157 (10)
C5	0.0624 (10)	0.0480 (11)	0.0518 (9)	0.0075 (8)	0.0268 (8)	0.0018 (7)
C6	0.0450 (8)	0.0503 (10)	0.0395 (7)	0.0137 (7)	0.0162 (6)	0.0063 (6)
C7	0.130 (3)	0.159 (3)	0.0798 (16)	0.037 (2)	0.0733 (18)	0.0119 (19)
C8	0.0398 (7)	0.0478 (10)	0.0487 (8)	0.0052 (7)	0.0201 (6)	0.0017 (7)
C9	0.0392 (7)	0.0441 (10)	0.0535 (8)	0.0035 (7)	0.0229 (6)	0.0005 (7)
C10	0.0375 (7)	0.0506 (10)	0.0490 (8)	0.0058 (7)	0.0159 (6)	−0.0033 (7)
C11	0.0623 (11)	0.0632 (13)	0.0556 (10)	0.0202 (9)	0.0273 (8)	0.0082 (9)
C12	0.0911 (16)	0.0768 (17)	0.0577 (11)	0.0305 (13)	0.0201 (10)	0.0146 (11)
C13	0.0655 (12)	0.0806 (18)	0.0706 (13)	0.0360 (12)	−0.0040 (10)	−0.0071 (12)
C14	0.0433 (10)	0.0823 (18)	0.1062 (18)	0.0183 (10)	0.0237 (11)	−0.0094 (14)
C15	0.0480 (10)	0.0686 (15)	0.0930 (14)	0.0100 (9)	0.0367 (10)	0.0057 (12)
C16	0.0595 (10)	0.0586 (12)	0.0638 (10)	0.0104 (9)	0.0351 (8)	0.0095 (9)

### Geometric parameters (Å, °)

**Table:**

S1—C8	1.6746 (18)	C7—H7A	0.9600
N1—N2	1.372 (2)	C7—H7B	0.9600
N1—C9	1.287 (2)	C7—H7C	0.9600
N2—C8	1.362 (2)	C9—C10	1.478 (2)
N2—H2	0.90 (2)	C9—C16	1.499 (2)
N3—C6	1.424 (2)	C10—C11	1.391 (3)
N3—C8	1.340 (2)	C10—C15	1.395 (2)
N3—H3	0.82 (2)	C11—H11	0.9300
C1—H1	0.9300	C11—C12	1.375 (3)
C1—C2	1.386 (3)	C12—H12	0.9300
C1—C6	1.386 (3)	C12—C13	1.393 (4)
C2—H2A	0.9300	C13—H13	0.9300
C2—C3	1.373 (4)	C13—C14	1.361 (4)
C3—C4	1.378 (4)	C14—H14	0.9300
C3—C7	1.517 (3)	C14—C15	1.376 (3)
C4—H4	0.9300	C15—H15	0.9300
C4—C5	1.388 (3)	C16—H16A	0.9600
C5—H5	0.9300	C16—H16B	0.9600
C5—C6	1.376 (3)	C16—H16C	0.9600
			
C9—N1—N2	118.83 (15)	N2—C8—S1	119.57 (13)
N1—N2—H2	126.2 (14)	N3—C8—S1	125.62 (12)
C8—N2—N1	118.67 (15)	N3—C8—N2	114.78 (16)
C8—N2—H2	114.3 (14)	N1—C9—C10	115.87 (15)
C6—N3—H3	117.8 (16)	N1—C9—C16	123.94 (16)
C8—N3—C6	126.88 (15)	C10—C9—C16	120.17 (14)
C8—N3—H3	115.0 (16)	C11—C10—C9	121.03 (15)
C2—C1—H1	120.3	C11—C10—C15	118.04 (18)
C2—C1—C6	119.4 (2)	C15—C10—C9	120.93 (17)
C6—C1—H1	120.3	C10—C11—H11	119.7
C1—C2—H2A	119.3	C12—C11—C10	120.6 (2)
C3—C2—C1	121.4 (2)	C12—C11—H11	119.7
C3—C2—H2A	119.3	C11—C12—H12	119.9
C2—C3—C4	118.58 (18)	C11—C12—C13	120.2 (2)
C2—C3—C7	121.2 (3)	C13—C12—H12	119.9
C4—C3—C7	120.2 (3)	C12—C13—H13	120.2
C3—C4—H4	119.5	C14—C13—C12	119.7 (2)
C3—C4—C5	121.0 (2)	C14—C13—H13	120.2
C5—C4—H4	119.5	C13—C14—H14	119.8
C4—C5—H5	120.1	C13—C14—C15	120.5 (2)
C6—C5—C4	119.86 (19)	C15—C14—H14	119.8
C6—C5—H5	120.1	C10—C15—H15	119.5
C1—C6—N3	121.13 (18)	C14—C15—C10	121.0 (2)
C5—C6—N3	119.00 (17)	C14—C15—H15	119.5
C5—C6—C1	119.75 (16)	C9—C16—H16A	109.5
C3—C7—H7A	109.5	C9—C16—H16B	109.5
C3—C7—H7B	109.5	C9—C16—H16C	109.5
C3—C7—H7C	109.5	H16A—C16—H16B	109.5
H7A—C7—H7B	109.5	H16A—C16—H16C	109.5
H7A—C7—H7C	109.5	H16B—C16—H16C	109.5
H7B—C7—H7C	109.5		
			
N1—N2—C8—S1	−172.72 (13)	C6—C1—C2—C3	−0.6 (3)
N1—N2—C8—N3	9.3 (2)	C7—C3—C4—C5	−179.6 (2)
N1—C9—C10—C11	−15.8 (3)	C8—N3—C6—C1	56.4 (3)
N1—C9—C10—C15	164.35 (19)	C8—N3—C6—C5	−127.6 (2)
N2—N1—C9—C10	−177.12 (15)	C9—N1—N2—C8	175.59 (16)
N2—N1—C9—C16	1.2 (3)	C9—C10—C11—C12	178.7 (2)
C1—C2—C3—C4	0.4 (3)	C9—C10—C15—C14	−178.5 (2)
C1—C2—C3—C7	179.7 (2)	C10—C11—C12—C13	0.3 (4)
C2—C1—C6—N3	176.51 (16)	C11—C10—C15—C14	1.6 (3)
C2—C1—C6—C5	0.6 (3)	C11—C12—C13—C14	0.6 (4)
C2—C3—C4—C5	−0.3 (3)	C12—C13—C14—C15	−0.4 (4)
C3—C4—C5—C6	0.3 (3)	C13—C14—C15—C10	−0.8 (4)
C4—C5—C6—N3	−176.47 (16)	C15—C10—C11—C12	−1.4 (3)
C4—C5—C6—C1	−0.4 (3)	C16—C9—C10—C11	165.88 (19)
C6—N3—C8—S1	3.6 (3)	C16—C9—C10—C15	−14.0 (3)
C6—N3—C8—N2	−178.56 (17)		

### Hydrogen-bond geometry (Å, °)

**Table:**

*D*—H···*A*	*D*—H	H···*A*	*D*···*A*	*D*—H···*A*
N2—H2···S1^i^	0.90 (2)	2.86 (2)	3.7456 (16)	167.8 (19)
C16—H16B···S1^i^	0.96	2.74	3.471 (2)	133

Symmetry codes: (i) −*x*+2, −*y*, −*z*+1.
